# Supplementary material for: Molecular and Crystal Structure of a Chitosan−Zinc Chloride Complex
Source: Nanomaterials (Basel). 2021 May 26;11(6):1407. doi: 10.3390/nano11061407 (PMC8229668; doi:10.3390/nano11061407)
Supplement: Supplementary file 1 [file nanomaterials-11-01407-s001.zip › nanomaterials-1235280-supplementary.pdf]

Supplementary Materials

Molecular and Crystal Structure of a Chitosan–Zinc Chloride Complex

Toshifumi Yui [1,*], Takuya Uto [2] and Kozo Ogawa [3]

[1] Faculty of Engineering, University of Miyazaki, Nishi 1-1 Gakuen-kibanadai, Miyazaki 889-2192, Japan; tyui@cc.miyazaki-u.ac.jp

[2] Organization for Promotion of Tenure Track, University of Miyazaki, Nishi 1-1 Gakuen-kibanadai, Miyazaki 889-2192, Japan; t.uto@cc.miyazaki-u.ac.jp

[3] Research Institute for Advanced Science and Technology, Osaka Prefecture University, 1-2 Gakuencho, Sakai, Osaka 599-8570, Japan; ogawakt@kawachi.zaq.ne.jp

* Correspondence: tyui@cc.miyazaki-u.ac.jp; Tel.: +81-985-58-7319

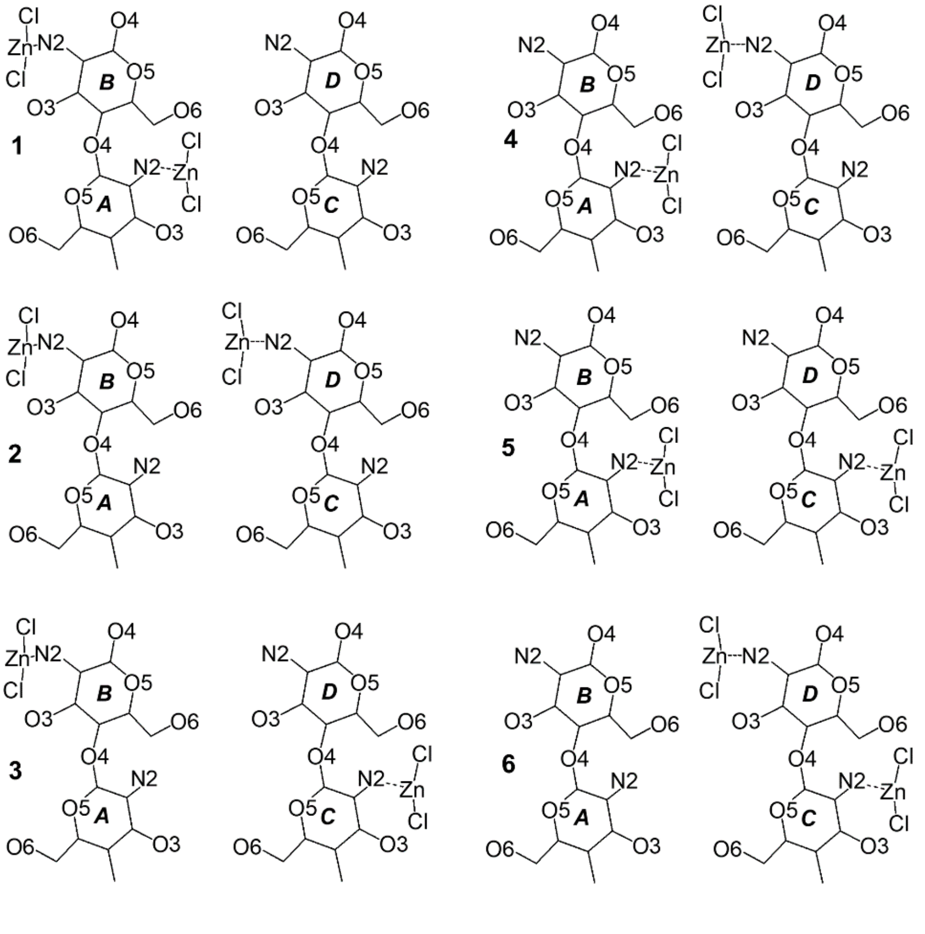

Figure S1. Six $ZnCl_2$ linking patterns for two independent chains.

Table S1. Calculated and observed *d*-spacings

hkl	$d_{calc.}$ (nm)	$d_{obsd.}$ (nm)	*hkl*	$d_{calc.}$ (nm)	$d_{obsd.}$ (nm)
100	1.1677	1.1816	113	0.3242	0.3252
020	0.8995	0.9106	203	0.2961	0.2952
120	0.7126	0.7182	143	0.2659	0.2620
200	0.5839	0.5861	233	0.2655	
220	0.4897	0.4896	333	0.2367	0.2375
050	0.3598		403	0.2225	
320	0.3572	0.3506	163	0.2218	0.2220
240	0.3563		413	0.2208	
400	0.2919				
160	0.2904	0.2902	104	0.2516	0.2509
410	0.2882		214	0.2337	0.2320
070	0.2570	0.2558	134	0.2320	
			044	0.2236	0.2240
011	0.8943	0.9117	054	0.2095	
111	0.7100	0.7337	324	0.2090	0.2093
211	0.4889	0.4949	244	0.2088	
131	0.4738	0.4601	064	0.1954	
141	0.3887	0.3966	344	01939	0.1942
231	0.3876		404	01932	
311	0.3569	0.3549			
331	0.3112	0.3131			
261	0.2582	0.2596			
102	0.4715	0.4760			
122	0.4176	0.4260			
032	0.3909	0.3931			
222	0.3550	0.3567			
142	0.3254	0.3212			
232	0.3248				
152	0.2860	0.2876			
342	0.2556	0.2551			

Table S2. R_w values of refined structures in the chain packing search

chain positions		chain polarities		hydroxymethyl conformation	R_w
chain 1 (u1, v1)	chain 2 (u2, v2)	chain 1	chain 2		
		$P2_1/a$ space group			
0.0, 0.0	0.0, 0.5	up	up	*gg*	0.335
				gt	0.358
				tg	0.351
		up	down	*gg*	0.310
				gt	0.289
				tg	0.290
0.25, 0.25	0.25, 0.75	up	up	*gg*	0.373
				gt	0.386
				tg	0.372
		up	down	*gg*	0.363
				gt	0.325
				tg	0.325
		$P2_1/b$ space group			
0.0, 0.0	0.5, 0.0	up	up	*gg*	0.497
				gt	0.470
				tg	0.482
		up	down	*gg*	0.497
				gt	0.459
				tg	0.466
0.25, 0.25	0.25, 0.75	up	up	*gg*	0.373
				gt	0.386
				tg	0.372
		up	down	*gg*	0.363
				gt	0.325
				tg	0.325

Table S3. Fractional atomic coordinates of the final crystal structure

	x	y	z		x	y	z
	Chain 1 (up)				Chain 3 (down)		
C1a	0.00969	0.01792	0.50121	C1a	0.02054	0.51353	-1.22949
C2a	0.07665	0.06534	0.40414	C2a	0.05672	0.57341	-1.13242
C3a	0.01622	0.06545	0.2722	C3a	0.08345	0.53823	-1.00048
C4a	-0.00449	-0.01448	0.2279	C4a	-0.01807	0.49101	-0.95618
C5a	-0.06884	-0.05722	0.33337	C5a	-0.04893	0.43471	-1.06165
C6a	-0.08421	-0.13927	0.29948	C6a	-0.1557	0.38968	-1.02776
O6a	-0.16707	-0.14519	0.19726	O6a	-0.12748	0.33877	-0.92554
O5a	-0.00039	-0.05505	0.44743	O5a	-0.07601	0.47557	-1.17571
N2a	0.08365	0.1424	0.45054	N2a	0.16029	0.61137	-1.17882
O3a	0.0906	0.09937	0.17852	O3a	0.09768	0.5965	-0.9068
O4a	-0.07598	-0.01233	0.11477	O4a	0.01635	0.45029	-0.84305
H1a	-0.07548	0.04275	0.52161	H1a	0.09234	0.47479	-1.24989
H2a	0.16485	0.04276	0.39241	H2a	-0.0133	0.6149	-1.12069
H3a	-0.06661	0.09599	0.278	H3a	0.16212	0.50337	-1.00628
H4a	0.07812	-0.04274	0.2058	H4a	-0.09349	0.52675	-0.93408
H5a	-0.15402	-0.03142	0.35155	H5a	0.02423	0.3964	-1.07983
H61a	-0.00202	-0.16416	0.26662	H61a	-0.22629	0.42666	-0.9949
H62a	-0.11463	-0.17086	0.38556	H62a	-0.18605	0.35806	-1.11384
C1b	-0.00935	-0.01787	0.00124	C1b	-0.02062	0.48669	-0.72952
C2b	-0.07647	-0.06529	-0.09569	C2b	-0.05674	0.42672	-0.63259
C3b	-0.01612	-0.06543	-0.22773	C3b	-0.08346	0.46184	-0.50055
C4b	0.0046	0.0145	-0.27207	C4b	0.01804	0.50905	-0.45621
C5b	0.06905	0.05723	-0.16668	C5b	0.04884	0.56541	-0.56161
C6b	0.08444	0.13927	-0.2006	C6b	0.1556	0.61045	-0.52768
O6b	0.16817	0.14523	-0.3019	O6b	0.12705	0.66188	-0.42638
O5b	0.00069	0.05508	-0.05255	O5b	0.07592	0.52462	-0.67573
N2b	-0.08341	-0.14236	-0.04927	N2b	-0.16033	0.3888	-0.67901
O3b	-0.09059	-0.09934	-0.32133	O3b	-0.09764	0.40352	-0.40695
O4b	0.076	0.01232	-0.38528	O4b	-0.01637	0.54972	-0.343
H1b	0.07584	-0.04275	0.02133	H1b	-0.0925	0.52542	-0.74961
H2b	-0.16467	-0.04268	-0.10732	H2b	0.01332	0.38524	-0.62096
H3b	0.0667	-0.09598	-0.22201	H3b	-0.16215	0.49668	-0.50627
H4b	-0.07801	0.04277	-0.2941	H4b	0.09348	0.47333	-0.43418
H5b	0.15424	0.03141	-0.14858	H5b	-0.02434	0.60372	-0.5797
H61b	0.00245	0.16402	-0.23445	H61b	0.2259	0.57354	-0.49383
H62b	0.114	0.17098	-0.11427	H62b	0.18649	0.64162	-0.61401
	Chain 2 (down)				Chain 4 (up)		
C1a	0.50969	-0.01792	-0.50121	C1a	0.52054	-0.51353	1.22949
C2a	0.57665	-0.06534	-0.40414	C2a	0.55672	-0.57341	1.13242
C3a	0.51622	-0.06545	-0.2722	C3a	0.58345	-0.53823	1.00048
C4a	0.49551	0.01448	-0.2279	C4a	0.48193	-0.49101	0.95618
C5a	0.43116	0.05722	-0.33337	C5a	0.45107	-0.43471	1.06165
C6a	0.41579	0.13927	-0.29948	C6a	0.3443	-0.38968	1.02776
O6a	0.33223	0.14524	-0.198	O6a	0.37276	-0.33835	0.92628
O5a	0.49961	0.05505	-0.44743	O5a	0.42399	-0.47557	1.17571
N2a	0.58365	-0.1424	-0.45054	N2a	0.66029	-0.61137	1.17882
O3a	0.5906	-0.09937	-0.17852	O3a	0.59768	-0.5965	0.9068
O4a	0.42402	0.01233	-0.11477	O4a	0.51635	-0.45029	0.84305

H1a	0.42452	-0.04275	-0.52161	H1a	0.59234	-0.47479	1.24989
H2a	0.66485	-0.04276	-0.39241	H2a	0.4867	-0.6149	1.12069
H3a	0.43339	-0.09599	-0.278	H3a	0.66212	-0.50337	1.00628
H4a	0.57812	0.04274	-0.2058	H4a	0.40651	-0.52675	0.93408
H5a	0.34598	0.03142	-0.35155	H5a	0.52423	-0.3964	1.07983
H61a	0.49782	0.16404	-0.26584	H61a	0.27395	-0.42661	0.99412
H62a	0.38607	0.17094	-0.38577	H62a	0.31353	-0.35843	1.11406
C1b	0.49065	0.01787	-0.00124	C1b	0.47938	-0.48669	0.72952
C2b	0.42353	0.06529	0.09569	C2b	0.44326	-0.42672	0.63259
C3b	0.48388	0.06543	0.22773	C3b	0.41654	-0.46184	0.50055
C4b	0.5046	-0.0145	0.27207	C4b	0.51804	-0.50905	0.45621
C5b	0.56905	-0.05723	0.16668	C5b	0.54884	-0.56541	0.56161
C6b	0.58444	-0.13927	0.2006	C6b	0.6556	-0.61045	0.52768
O6b	0.679	-0.16807	0.12735	O6b	0.65389	-0.67823	0.60093
O5b	0.50069	-0.05508	0.05255	O5b	0.57592	-0.52462	0.67573
N2b	0.41659	0.14236	0.04927	N2b	0.33967	-0.3888	0.67901
O3b	0.40941	0.09934	0.32133	O3b	0.40236	-0.40352	0.40695
O4b	0.576	-0.01232	0.38528	O4b	0.48363	-0.54972	0.343
H1b	0.57584	0.04275	-0.02133	H1b	0.4075	-0.52542	0.74961
H2b	0.33533	0.04268	0.10732	H2b	0.51332	-0.38524	0.62096
H3b	0.5667	0.09598	0.22201	H3b	0.33785	-0.49668	0.50627
H4b	0.42199	-0.04277	0.2941	H4b	0.59348	-0.47333	0.43418
H5b	0.65424	-0.03141	0.14858	H5b	0.47566	-0.60372	0.5797
H61b	0.60215	-0.14644	0.30586	H61b	0.65775	-0.62393	0.42242
H62b	0.50567	-0.1711	0.17641	H62b	0.73429	-0.57853	0.55187
	$ZnCl_2$-1				$ZnCl_2$-3		
Zn	0.28277	0.27764	0.74037	Zn	0.2599	0.7869	-1.46865
Cl1	0.32541	0.31628	0.92798	Cl1	0.29463	0.82875	-1.65626
Cl2	0.17795	0.29067	0.55594	Cl2	0.32402	0.73152	-1.28422
	$ZnCl_2$-2				$ZnCl_2$-4		
Zn	-0.10869	-0.25864	0.33621	Zn	-0.31013	0.32293	-1.06449
Cl1	-0.02315	-0.2839	0.16087	Cl1	-0.3827	0.36169	-0.88915
Cl2	-0.29808	-0.28504	0.4279	Cl2	-0.26341	0.20092	-1.15618

Table S4. Observed and calculated structure factor amplitudes of the final structure

spot no.	*h*	*k*	*l*	F_{calc}	F_{obs}
1	0	1	0	38.61	(41.59)[a]
2	1	0	0	27.9	73.07
3	1	-1	0	79.69	(57.06)
	1	1	0		
4	0	2	0	139.2	139.18
5	1	-2	0	225.87	351.57
	1	2	0		
6	0	3	0	63.57	(75.1)
7	2	0	0	174.4	107.67
8	2	-1	0	127.5	(78.65)
	2	1	0		
9	1	-3	0	88.29	(80.62)
	1	3	0		
10	2	-2	0	503.25	496.21
	2	2	0		
11	0	4	0	101.13	(89.97)
12	1	-4	0	214.9	(94.46)
	1	4	0		
	2	-3	0		
	2	3	0		
13	3	0	0	136.06	(99.57)
14	3	-1	0	107.86	(101.25)
	3	1	0		
15	0	5	0	424.55	482.04
	3	-2	0		
	3	2	0		
	2	-4	0		
	2	4	0		
16	1	-5	0	63.58	(77.38)
	1	5	0		
17	3	-3	0	92.15	(80.75)
	3	3	0		
18	2	-5	0	34.27	(85.32)
	2	5	0		
19	0	6	0	128.16	(87.73)
	3	-4	0		
	3	4	0		
20	4	0	0	244.79	247.07
	1	-6	0		
	1	6	0		
	4	-1	0		
	4	1	0		
21	4	-2	0	83.66	(93.49)
	4	2	0		
22	2	-6	0	140.56	(97.37)
	2	6	0		
23	3	-5	0	130.87	(98.65)
	3	5	0		
	4	-3	0		
	4	3	0		
24	0	7	0	110.79	157.63
23	4	-3	0	130.87	(98.65)
	4	3	0		
24	0	7	0	110.79	157.63

spot no.	*h*	*k*	*l*	F_{calc}	F_{obs}
44	3	-4	1	34.29	(89.9)
	3	4	1		
45	4	0	1	155.28	(90.81)
	1	-6	1		
	1	6	1		
46	4	-1	1	75.66	(91.7)
	4	1	1		
47	4	-2	1	113.59	(95.05)
	4	2	1		
48	2	-6	1	176	199.25
	2	6	1		
49	0	1	2	67.09	(30.11)
50	1	0	2	78.9	75.2
51	1	-1	2	95.31	(42.37)
	1	1	2		
52	0	2	2	35.15	(44.43)
53	1	-2	2	108.13	154.97
	1	2	2		
54	0	3	2	70.33	125.81
55	2	0	2	131.51	(57.09)
56	2	-1	2	84.7	(58.86)
	2	1	2		
57	1	-3	2	78.22	(60.38)
	1	3	2		
58	2	-2	2	172.72	210.32
	2	2	2		
59	0	4	2	51.6	(67.48)
60	1	-4	2	177.95	195.56
	1	4	2		
	2	-3	2		
	2	3	2		
61	3	0	2	34.87	(74.79)
62	3	-1	2	133.88	(76.07)
	3	1	2		
63	0	5	2	55.83	(79.42)
64	3	-2	2	69.58	(79.76)
	3	2	2		
	2	-4	2		
	2	4	2		
65	1	-5	2	133.86	124.08
	1	5	2		
66	3	-3	2	76.77	(85.97)
	3	3	2		
67	2	-5	2	113.21	(90.92)
	2	5	2		
68	0	6	2	85.19	(92.69)
69	3	-4	2	129.68	189.07
	3	4	2		
70	0	1	3	23.3	(32.53)
71	1	0	3	42.2	(36.53)
72	1	-1	3	129.05	130.55
	1	1	3		
73	0	2	3	81.38	(44.88)

25	0	1	1	126.72	88.45
26	1	0	1	63.05	(37.36)
27	1	-1	1	91.25	117.47
	1	1	1		
28	0	2	1	68.21	(42.89)
29	1	-2	1	116.3	(48.78)
	1	2	1		
30	0	3	1	86.42	(53.92)
31	2	0	1	77.54	(54.79)
32	2	-1	1	165.47	156.15
	2	1	1		
33	1	-3	1	95.9	145.07
	1	3	1		
	2	-2	1		
34	2	2	1	126.3	(61.09)
35	0	4	1	56.48	(64.62)
36	1	-4	1	180.95	147.54
	1	4	1		
	2	-3	1		
	2	3	1		
37	3	0	1	70.67	(71.49)
38	3	-1	1	100.04	110.8
	3	1	1		
39	0	5	1	122.39	(76.19)
	3	-2	1		
	3	2	1		
	2	-4	1		
	2	4	1		
40	1	-5	1	119.55	(78.62)
	1	5	1		
41	3	-3	1	168.02	191.43
	3	3	1		
42	2	-5	1	66.09	(86.7)
	2	5	1		
43	0	6	1	42.34	(88.38)

74	1	-2	3	74.87	(52.69)
	1	2	3		
75	0	3	3	65	(59.11)
76	2	0	3	126.08	178.88
77	2	-1	3	93.67	(62.2)
	2	1	3		
78	1	-3	3	82.99	(63.08)
	1	3	3		
79	2	-2	3	115.02	(67.78)
	2	2	3		
80	0	4	3	89.87	(72)
81	1	-4	3	130.22	145.48
	1	4	3		
	2	-3	3		
	2	3	3		
82	3	0	3	14.69	(80.16)
83	3	-1	3	113.24	(80.88)
	3	1	3		
84	0	5	3	116.88	(85.74)
	3	-2	3		
	3	2	3		
	2	-4	3		
	2	4	3		
85	1	-5	3	110.02	(88.63)
	1	5	3		
86	3	-3	3	220.07	247.22
	3	3	3		
87	2	-5	3	105.84	(98.21)
	2	5	3		
88	0	6	3	81.43	(99.2)
89	3	-4	3	175.93	(102)
	3	4	3		
90	4	0	3	258.47	260.95
	1	-6	3		
	1	6	3		

[a] Reflections with F_{obs} values in parenthesis are those for unobserved reflections.
